# Patient-reported outcomes in individuals with advanced gastrointestinal stromal tumor treated with ripretinib in the fourth-line setting: analysis from the phase 3 INVICTUS trial

**DOI:** 10.1186/s12885-022-10379-9

**Published:** 2022-12-13

**Authors:** Patrick Schöffski, Suzanne George, Michael C. Heinrich, John R. Zalcberg, Sebastian Bauer, Hans Gelderblom, César Serrano, Robin L. Jones, Steven Attia, Gina D’Amato, Ping Chi, Peter Reichardt, Claus Becker, Kelvin Shi, Julie Meade, Rodrigo Ruiz-Soto, Jean-Yves Blay, Margaret von Mehren

**Affiliations:** 1 General Medical Oncology, University Hospitals Leuven, Herestraat 49, B-3000 Leuven, Belgium; 2 Dana-Farber Cancer Institute, 450 Brookline Ave, 02215 Boston, MA USA; 3 VA Portland Veterans Health Care System, 3710 SW US Veterans Hospital Rd., 97239 Portland, OR USA; 4 OHSU Knight Cancer Institute, 3161 SW Pavilion Loop, 97239 Portland, OR USA; 5 Monash University and Alfred Health, 553 St Kilda Road, VIC 3004 Melbourne, Australia; 6 Department of Medical Oncology, University Hospital Essen, Sarcoma Center/West German Cancer Center, University Duisburg-Essen, Hufelandstraße 55, D – 45147 Essen, Germany; 7 German Cancer Consortium (DKTK), Partner site University Hospital Essen, Essen, Germany; 8 Leiden University Medical Center, Albinusdreef 2, 2333 ZA Leiden, Netherlands; 9 Centro Cellex, Vall d’Hebron Institute of Oncology, Carrer de Natzaret, 115-117, 08035 Barcelona, Spain; 10 Royal Marsden and Institute of Cancer Research, 15 Cotswold Rd, SM2 5NG London, UK; 11 Mayo Clinic, 4500 San Pablo Road S, 32224 Jacksonville, FL USA; 12 Sylvester Comprehensive Cancer Center, University of Miami, 1475 NW 12th Ave, 33136 Miami, FL USA; 13 Memorial Sloan Kettering Cancer Center, 1275 York Ave, 10065 New York, NY USA; 14 Sarcoma Center, Helios Klinikum Berlin-Buch, Schwanebecker Ch 50, 13125 Berlin, Germany; 15 Deciphera Pharmaceuticals, LLC, 200 Smith St., 02451 Waltham, MA USA; 16 Centre Leon Berard, 28 Prom. Léa et Napoléon Bullukian, 69008 Lyon, France; 17 Fox Chase Cancer Center, 333 Cottman Ave, 19111 Philadelphia, PA USA

**Keywords:** Gastrointestinal stromal tumors, Ripretinib, Patient-reported outcome measures, Quality of life, Alopecia

## Abstract

**Background:**

Ripretinib is a novel switch-control kinase inhibitor that inhibits KIT and PDGFRA signaling. In the INVICTUS phase 3 trial, ripretinib increased median progression-free survival and prolonged overall survival vs. placebo in ≥ fourth-line advanced GIST. Here, we report prespecified analysis of quality of life (QoL) as assessed by patient-reported outcome (PRO) measures and an exploratory analysis evaluating the impact of alopecia on QoL.

**Methods:**

In the INVICTUS trial (NCT03353753), QoL was assessed using the European Organisation for Research and Treatment of Cancer Quality of Life Questionnaire (EORTC QLQ-C30; physical function, role function, overall health, and overall QoL) and the EuroQoL 5-Dimension 5-Level (EQ-5D-5 L; visual analogue scale). Analysis of covariance (ANCOVA) models compared changes in scores from baseline to treatment cycle 2, day 1 within and between ripretinib and placebo. Within the ripretinib arm, repeated measures models assessed the impact of alopecia on QoL.

**Results:**

Patients receiving ripretinib maintained QoL (as assessed by the EORTC QLQ-C30 and EQ-5D-5 L PRO measures) from baseline to cycle 2, day 1 whereas QoL declined with placebo, resulting in clinically significant differences between treatments (nominal *P* < 0.01). The most common treatment-emergent adverse event with ripretinib was alopecia; however, QoL was similarly maintained out to treatment cycle 10, day 1 in patients receiving ripretinib who developed alopecia and those who did not.

**Conclusion:**

PRO assessments in the INVICTUS trial suggest that patients on ripretinib maintain their QoL out to C2D1, unlike patients receiving placebo. Longitudinal QoL was maintained for patients receiving ripretinib out to cycle 10, day 1 (approximately 8 months; past the point of median progression-free survival with ripretinib [6.3 months]), even if the patients developed alopecia.

**Trial registration:**

ClinicalTrials.gov Identifier: NCT03353753; first posted: November 27, 2017.

**Supplementary Information:**

The online version contains supplementary material available at 10.1186/s12885-022-10379-9.

## Introduction

Gastrointestinal stromal tumor (GIST) is the most common sarcoma of the gastrointestinal tract, with an estimated incidence of 10–15 cases per million [1, 2]. Activating mutations in KIT or platelet-derived growth factor receptor alpha (PDGFRA) occur in approximately 82–87% of patients with GIST [3], making these tumors amenable to treatment with tyrosine kinase inhibitors (TKIs). There are currently five approved TKIs (imatinib, sunitinib, regorafenib, ripretinib and avapritinib) for the treatment of patients with GIST.

While these TKIs have a more tolerable safety profile relative to traditional chemotherapy, they are associated with specific treatment-emergent adverse events (TEAEs) with potential quality of life (QoL) implications, such as various skin toxicities [4–8]. Palmar-plantar erythrodysesthesia syndrome (PPES), as well as stomatitis, are common with TKI therapy, especially with sunitinib and regorafenib; even Grade 2 severity of these common TEAEs can have significant impact on activities of daily living [4, 8–10]. Therefore, it is critical to evaluate patient-reported outcome (PRO) measures in patients with GIST receiving TKI therapy. In a systematic review of the current literature on TKI therapy in GIST, only 13 of 104 studies evaluated health-related QoL; common issues negatively affecting QoL were severe fatigue and fear of recurrence or progression of disease [11–13]. Most patients treated with sunitinib reported a decrease in health-related QoL whereas patients reported mostly stable QoL while receiving other TKIs [13].

Ripretinib, a novel switch-control TKI designed to broadly inhibit KIT and PDGFRA kinase signaling through a dual mechanism of action [14], is approved for the treatment of adult patients with advanced GIST who have received prior treatment with three or more TKIs, including imatinib, based on the results of the phase 3 INVICTUS study (NCT03353753) [15, 16]. In INVICTUS, ripretinib demonstrated a significant improvement in median progression-free survival (PFS), the primary endpoint, vs. placebo (6.3 vs. 1.0 months, respectively; hazard ratio [HR] = 0.15; 95% confidence interval [CI], 0.09‒0.25; *P* < 0.0001), with clinically meaningful prolongation of median overall survival ([OS], 15.1 vs. 6.6 months; HR = 0.36; 95% CI, 0.21–0.62) and an acceptable safety profile. Alopecia was the most common drug-related TEAE reported in patients taking ripretinib (49%) [16].

Here, we further describe the prespecified PRO assessments from patients receiving ripretinib or placebo from the INVICTUS trial and additional exploratory analyses of the impact of alopecia on self-reported functioning, health status, and QoL.

## Materials and methods

### Study design and participants

INVICTUS is an international, multicenter, randomized, double-blind, placebo-controlled phase 3 trial in 129 patients who received at least three prior TKIs for advanced GIST (Fig. S1). The study design has been previously described [16]. Key inclusion criteria included age ≥ 18 years with a diagnosis of GIST with at least one measurable lesion according to modified Response Evaluation Criteria In Solid Tumors (mRECIST) version 1.1 and prior progression on at least imatinib, sunitinib, and regorafenib or intolerance despite dose modifications. Patients were randomized 2:1 to receive ripretinib 150 mg once daily (*n* = 85) or placebo (*n* = 44) until progressive disease (PD) or unacceptable toxicity. PD was determined by blinded independent central review using mRECIST version 1.1. Patients randomized to placebo could cross over to ripretinib 150 mg once daily at the time of confirmed PD and patients who progressed on ripretinib 150 mg once daily could dose-escalate to ripretinib 150 mg twice daily, continue treatment at the same dose, or discontinue treatment [17, 18]. Treatment cycles were 28 days. The protocol for the INVICTUS study is published online [19]. This analysis only includes PRO assessments from patients conducted during the placebo-controlled, double-blind portion of the trial (data cutoff: May 31, 2019).

### PRO assessments

PRO assessments consisted of the European Organisation for Research and Treatment of Cancer Quality of Life Questionnaire (EORTC QLQ-C30) and the EuroQoL 5-Dimension 5-Level (EQ-5D-5 L). These were completed using an electronic PRO (ePRO) system before dosing on days 1 (baseline) and 15 of cycle 1, day 1 of subsequent cycles, and within 7 days of the last dose (end-of-treatment visit).

The EQ-5D-5 L [20] and EORTC QLQ-C30 [21] are both validated, standardized, patient-completed questionnaires used extensively in cancer clinical studies, for which validated translations were provided for sites in non-English-speaking countries (Table 1). The EQ-5D-5 L was developed by the EuroQoL group to provide a measure of patient utility for clinical and economic appraisals [20] and includes the EQ-5D-5 L visual analogue scale (VAS). Patients used the EQ-5D-5 L VAS to rate their health on that specific day on a vertical scale. The EORTC QLQ-C30, developed to assess health-related QoL in patients with cancer, is composed of multi-item and single-item scales, including functioning scales (physical functioning and role functioning), and a global health status/QoL scale [21]. The physical functioning and role functioning scales are two of the PROs identified by the US Food and Drug Administration as existing tools that measure core PROs that are clinically relevant and important to patients [22]. The functioning scales asked the patients to rate their experiences for seven items during the previous week on a four-point scale. Two items comprising the global health status scale also evaluated the patient’s experience over the past week. The two functional scales (EORTC QLQ-C30 physical and role function) and the self-reported health status (EQ-5D-5 L VAS) were prespecified because they illuminate critical aspects of the patient experience not covered otherwise. The additional function and symptom scales of the EORTC QLQ-C30 were not prespecified analyses to avoid alpha-split issues. Additional data from the PRO questionnaires (including symptom items) are presented in the Supplementary information (Tables S1 and S2). In scoring the EORTC QLQ-C30, the overall health and QoL scales may be combined into a single score [23]. However, since the scales measure fundamentally different aspects of the patient experience, one could improve while the other deteriorates. Thus, reporting the average of the two scores would be problematic and is not done in the prespecified analysis.

**Table 1:** Patient-reported outcome assessments

Patient-reported outcomes	Description
**EQ-5D-5 L [**20**]**	
**Visual analogue scale**	• Records self-rated health on a vertical VAS • “We would like to know how good or bad your health is TODAY” • Ranges from 0 (worst imaginable state of health) to 100 (best imaginable state of health)
**EORTC QLQ-C30 [**21**]**	
**Physical function**	• Five questions evaluating strength, endurance, and daily physical functioning • Four-point rating scale ranging from “1-not at all” to “4-very much” • Responses were rolled up to a score ranging from 0 to 100 in which a larger value is better, per the EORTC manual [23]
**Role function**	• Two questions evaluating limitations during everyday activities • Four-point rating scale ranging from “1-not at all” to “4-very much” • Responses were rolled up to a score ranging from 0 to 100 in which a larger value is better, per the EORTC manual [23]
** Overall health (question C29)**^**a**^	• One question asking patients to rate their overall health during the past week on a scale of 1 (very poor) to 7 (excellent)
** Overall quality of life (question C30)**^**a**^	• One question asking patients to rate their overall quality of life during the past week on a scale of 1 (very poor) to 7 (excellent)

*EORTC QLQ-C30* European Organisation for the Research and Treatment of Cancer Quality of Life Questionnaire, *EQ-5D-5 L* EuroQoL 5-Dimension 5-Level, *VAS* Visual analogue scale

^**a**^Questions C29 and C30 were additional analyses; the three other analyses were prespecified

Changes from baseline in PRO measures were assessed using minimal clinically important difference (MCID) as identified in a review of MCID values for GI-related cancers [24]. For the EQ-5D-5 L VAS, MCID ranged from 7–8 points for anchor-based estimates and 9–11 points for distribution-based estimates from a retrospective analysis of patients with various types of cancers [25]. For the EORTC QLQ-C30 functioning scales and health-related QoL, there have been no empirically drawn MCIDs for GI-related cancers; however, an analysis encompassing several different conditions found that the MCID was approximately 0.5 times the standard deviation of the baseline value [26].

### Safety assessments

TEAEs were monitored throughout the study from informed consent to safety follow-up, occurring 30 days after the last dose, with their severity rated by investigators using the National Cancer Institute Common Terminology Criteria for Adverse Events (NCI-CTCAE) version 4.03 [16]. Time to first appearance and time to worst grade of the TEAE were recorded.

### Statistical analyses

The EORTC QLQ-C30 was summarized by scale. In the scales used in the current manuscript, a higher score reflects better functioning or perceptions of overall health/QoL. The scoring for this questionnaire was done in two steps, with initial calculation of the average of the items that contribute to the scale. This was used as the raw score for the scale, to which a linear transformation was applied to standardize it, so that scores ranged from 0 to 100. The EQ-5D-5 L VAS was summarized using continuous descriptive statistics.

Statistical comparisons between treatment arms were carried out on cycle 2, day 1 (C2D1; prespecified endpoint) for two functioning scales and the EQ-5D-5 L VAS. The number of patients in the placebo arm was low after this point due to attrition. The PRO-evaluable population includes patients with available baseline and C2D1 assessments; patients who did not have both a baseline and a C2D1 value were dropped from the analysis and no imputation of missing values was done. For selected domains from the EORTC QLQ-C30 (physical function, role function, overall health, overall QoL), analysis of covariance (ANCOVA) models were built for change from baseline to C2D1, with the stratification factors as factors. Fixed effects were treatment, Eastern Cooperative Oncology Group (ECOG) score at baseline, and the number of prior anticancer treatments. For the EQ-5D-5 L VAS, a t-test was performed between the ripretinib and placebo group for their change from baseline to C2D1 scores.

In exploratory analyses, generalized estimating equation models were created to compare patients with and without alopecia. Models were built for each of the five PROs for ripretinib patients using repeated measures models across visits. For patients with alopecia, cycles 1 and 2 were excluded to account for the median time of alopecia onset. Covariates were sex, alopecia (yes/no), and ECOG score at baseline. When there was no end date available for the TEAE, the event was coded conservatively as having extended to the last visit of the double-blind period. Due to the placement of PROs in the hierarchy of testing, all *P*-values reported in this article are nominal.

## Results

### Patient population

**Fig. 1:**
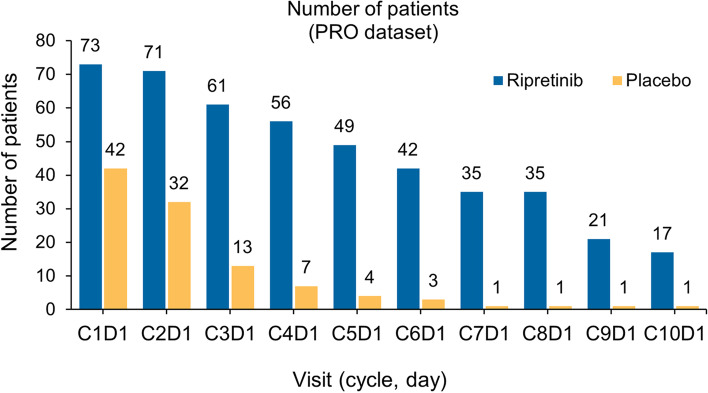
Number of patients with PRO assessment over time. At C1D1, 73 patients were evaluable in the ripretinib arm, and 42 patients were evaluable in the placebo arm. C, cycle; D, day; PRO, patient-reported outcome

Of 84 patients who received ripretinib during the double-blind period, the PRO-evaluable population included 73 patients with an available baseline assessment and 71 patients with an available C2D1 assessment. Of the 43 patients who received placebo during the double-blind period, the PRO-evaluable population included 42 patients with an available baseline assessment and 32 patients with an available C2D1 assessment (Fig. 1). The declining number of patients with PRO data over time reflects the number of patients who remained progression-free as previously reported [16].

### Changes in EQ-5D-5 L VAS and EORTC QLQ-C30 PRO measures

**Fig. 2:**
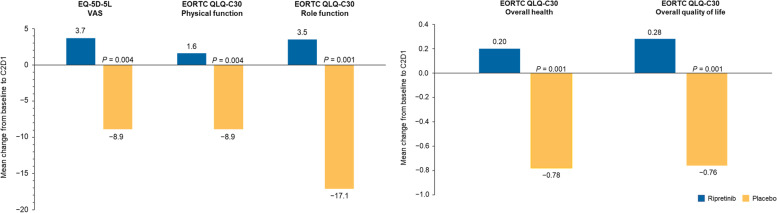
Change from baseline to cycle 2, day 1 in EQ-5D-5 L VAS and EORTC QLQ-C30 measures. Mean change from baseline to C2D1 in the EQ-5D-5 L VAS (A), EORTC QLQ-C30 physical function (A), EORTC QLQ-C30 role function (A), EORTC QLQ-C30 overall health (B), and EORTC QLQ-C30 quality of life (B). *P*-values are nominal, and no statistical significance is being claimed. The Physical and Role Function questions were rolled up to a score out of 100; questions C29 and C30 are based on 7-point scales. C2D1, cycle 2, day 1; EORTC QLQ-C30, European Organisation for the Research and Treatment of Cancer Quality of Life Questionnaire; EQ-5D-5 L, EuroQoL 5-Dimension 5-Level; VAS, visual analogue scale

**Fig. 3:**
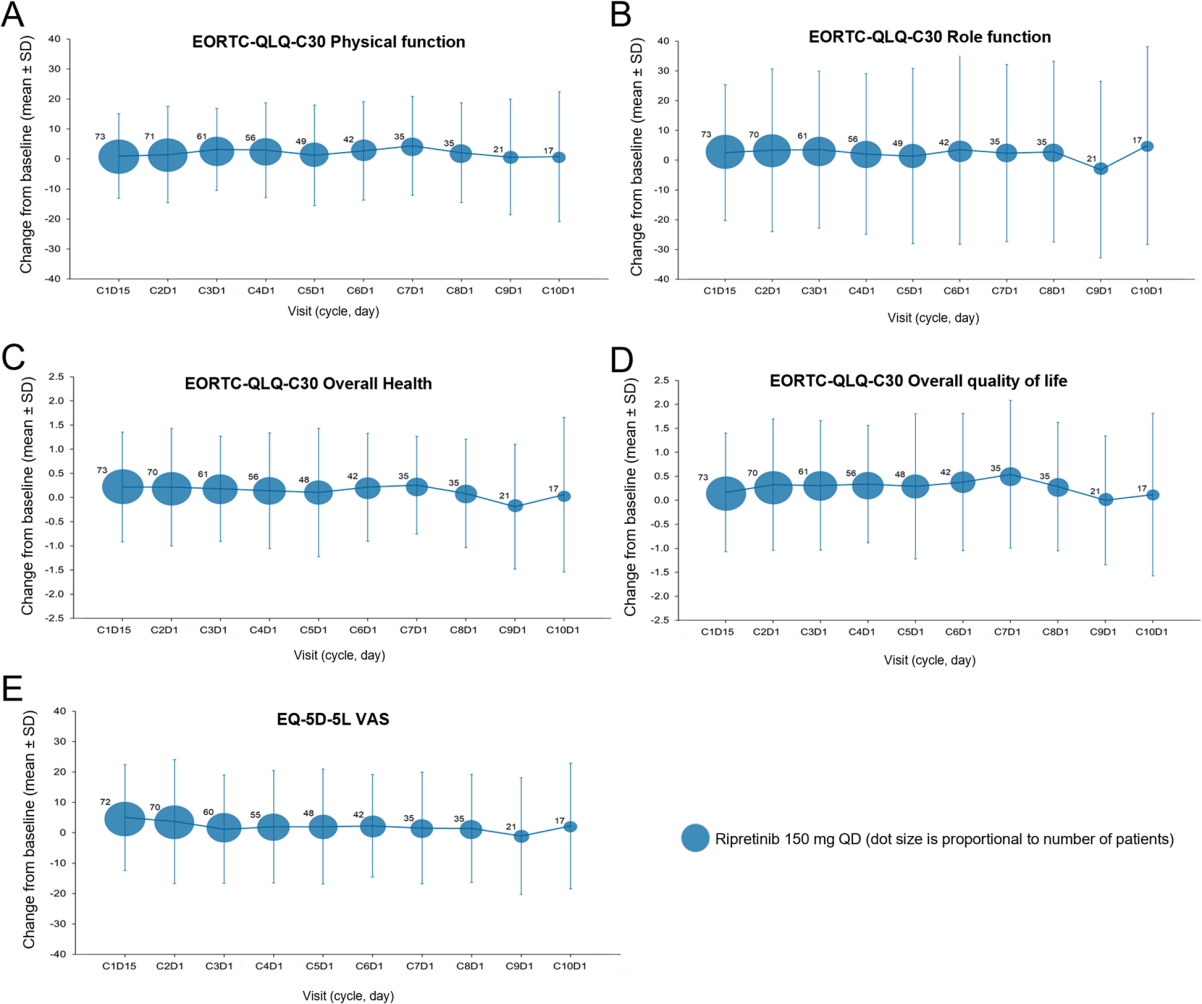
Longitudinal changes from baseline in EORTC QLQ-C30 measures and EQ-5D-5 L VAS in the ripretinib arm. Longitudinal change from baseline in EORTC QLQ-C30 physical function (**A**), role function (**B**), overall health (**C**), overall quality of life (**D**), and EQ-5D-5 L VAS (**E**). EORTC QLQ-C30, European Organisation for the Research and Treatment of Cancer Quality of Life Questionnaire; EQ-5D-5 L, EuroQoL 5-Dimension 5-Level; QD, once daily; SD, standard deviation; VAS, visual analogue scale

Changes from baseline to C2D1 in EQ-5D-5 L VAS and EORTC QLQ-C30 PRO measures are presented in Fig. 2. Patients receiving ripretinib maintained their daily self-reported health on the EQ-5D-5 L VAS, while placebo treatment was associated with a decline (nominal *P* = 0.004 for the difference between arms). Patients receiving ripretinib reported stable physical and role functioning relative to baseline, and the same measures deteriorated in patients receiving placebo (nominal *P* = 0.004 and nominal *P* = 0.001, respectively). Patients also maintained stable perceptions of their overall health and QoL compared with the placebo arm (both nominal *P* = 0.001). All differences between treatment arms exceeded the MCID. Scores at baseline and C2D1 for all items that were not part of the prespecified analysis are presented in the Supplementary information (Tables S1 and S2). Longitudinal changes in PRO scores from baseline in the ripretinib arm (Fig. 3) show that patients receiving ripretinib reported stable role and physical function, health status, and health QoL out to cycle 10, day 1 (approximately 8 months), which exceeds the previously reported median progression-free survival (6.3 months) [16].

### Safety

TEAEs (all causality) reported in > 20% of patients receiving ripretinib are summarized in Table 2 (data cut: May 31, 2019); the most common TEAE in the ripretinib arm was alopecia (*n* = 44, 52%). The incidence of alopecia was 57% in females and 43% in males. NCI-CTCAE grading for alopecia consists of two severity options: Grade 1 (< 50% hair loss) or Grade 2 (≥ 50% hair loss). The majority (34/44; 77%) of patients receiving ripretinib who developed alopecia had a severity of Grade 1 (data not shown).

**Table 2:** TEAEs in > 20% of patients receiving ripretinib^a^

Preferred term, n (%)	Ripretinib (*n* = 85)		Placebo (*n* = 43)	
	**All grades**	**Grade 3–4**	**All grades**	**Grade 3–4**
Alopecia	44 (52)	N/A	2 (4.7)	N/A
Fatigue	36 (42)	3 (3.5)	10 (23)	1 (2.3)
Nausea	33 (39)	3 (3.5)	5 (12)	0
Abdominal pain	31 (37)	6 (7.1)	13 (30)	2 (4.7)
Constipation	29 (34)	1 (1.2)	8 (19)	0
Myalgia	27 (32)	1 (1.2)	5 (12)	0
Diarrhea	24 (28)	1 (1.2)	6 (14)	1 (2.3)
Decreased appetite	23 (27)	1 (1.2)	9 (21)	1 (2.3)
PPES^b^	18 (21)	0	0	0
Vomiting	18 (21)	3 (3.5)	3 (7.0)	0

*N/A* Not applicable, *PPES* Palmar-plantar erythrodysesthesia syndrome, *TEAE* Treatment-emergent adverse event

^a^Includes all TEAEs regardless of drug relatedness

^b^The highest severity classification for PPES is Grade 3

### Emergence of alopecia and the association with QoL

**Fig. 4:**
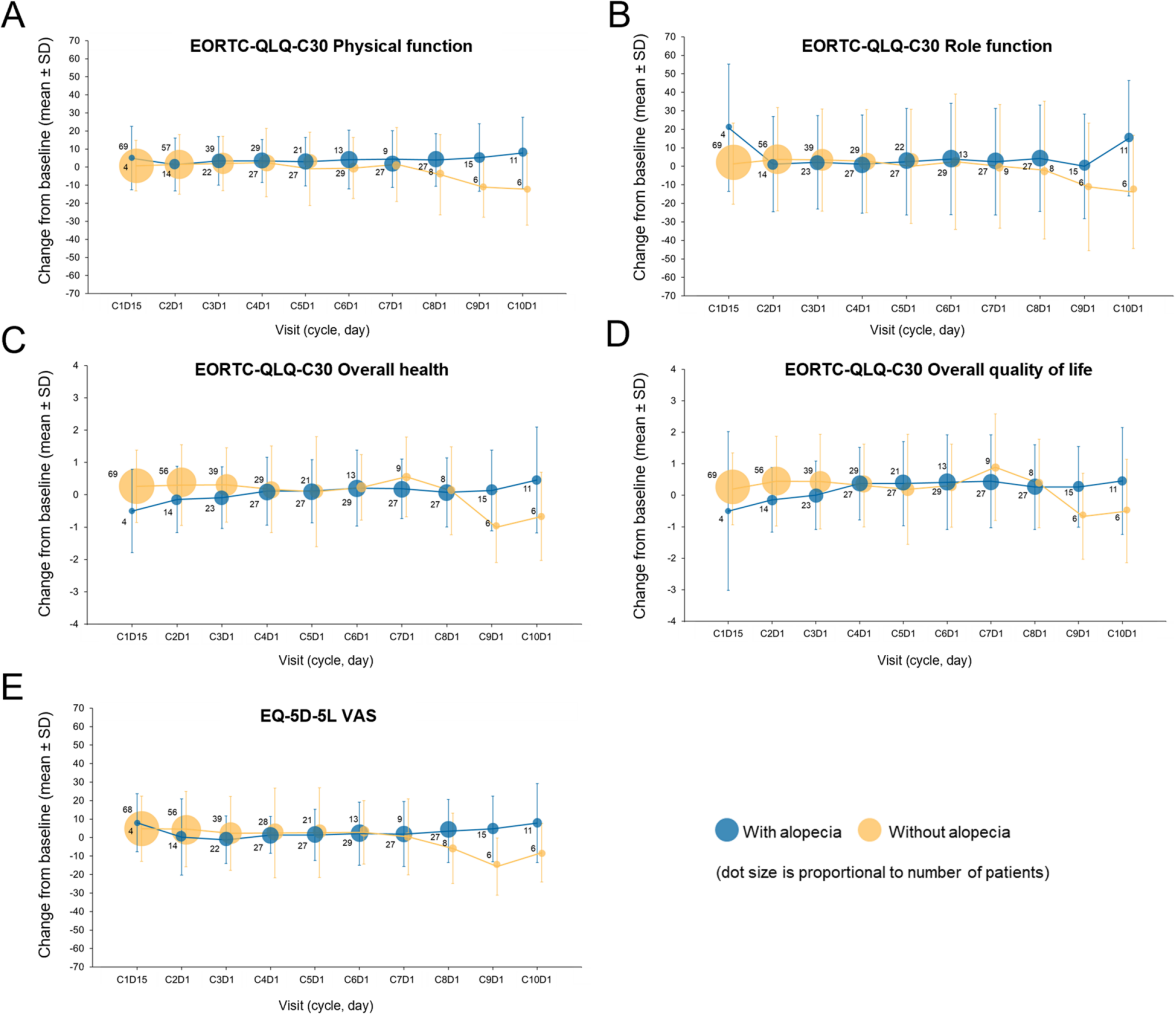
Longitudinal changes from baseline in PRO measures in patients with or without alopecia who received ripretinib. EORTC QLQ-C30 physical function (**A**), EORTC QLQ-C30 role function (**B**), EORTC QLQ-C30 overall health (**C**), EORTC QLQ-C30 quality of life (**D**), and EQ-5D-5 L VAS (**E**). EORTC QLQ-C30, European Organisation for the Research and Treatment of Cancer Quality of Life Questionnaire; EQ-5D-5 L, EuroQoL 5-Dimension 5-Level; SD, standard deviation; VAS, visual analogue scale

In patients receiving ripretinib, the median times to first appearance and to worst grade alopecia were 1.9 and 2.1 months, respectively. Repeated measures analysis (Table 3) showed that overall health and physical and role function were similarly maintained in patients receiving ripretinib with and without alopecia over time. A trend toward better self-reported overall QoL was observed for patients who experienced alopecia (compared with no alopecia; nominal *P* = 0.03); however, the difference did not exceed the threshold for meaningful change. Longitudinal graphs from cycle 1, day 1 out to cycle 10, day 1 demonstrate that responses for overall health, physical and role functioning, and overall QoL (average mean change from baseline) are generally maintained for patients receiving ripretinib who developed alopecia and those who did not (Fig. 4).

**Table 3:** General estimating equation analysis summary of the association between alopecia and the 5 PRO measures in patients taking ripretinib

	Mean estimate^a^	Confidence interval	*P*-value^b^
**Alopecia**			
EORTC QLQ-C30			
Overall health	0.17	(− 0.10, 0.44)	0.22
Overall quality of life	0.35	(0.03, 0.67)	0.03
Physical function	3.17	(− 0.29, 6.64)	0.07
Role function	4.50	(− 2.87, 11.87)	0.23
EQ-5D-5 L			
VAS	3.01	(− 0.64, 6.67)	0.11

*ECOG* Eastern Cooperative Oncology Group, *EORTC QLQ-C30* European Organisation for the Research and Treatment of Cancer Quality of Life Questionnaire, *EQ-5D-5 L* EuroQoL 5-Dimension 5-Level, *PRO* Patient-reported outcome, *VAS* Visual analogue scale

^a^Indicates the impact on PRO score in patients receiving ripretinib with alopecia vs. patients receiving ripretinib without alopecia, while keeping other variables constant (i.e., gender and ECOG status)

^b^All *P*-values reported are nominal

## Discussion

In the INVICTUS phase 3 study, ripretinib demonstrated a statistically and clinically significant improvement in PFS and a clinically meaningful OS benefit compared with placebo that has led to the approval of ripretinib in the US, Canada, Australia, Hong Kong, China, Taiwan, Switzerland, the UK, and the EU [16, 27–34]. Here, we show that all five key PRO measures were maintained out to C2D1 in patients with advanced GIST receiving ripretinib compared with declining measures reported with placebo. The differences between the two arms in physical function, role function, health, and QoL as rated by patients receiving ripretinib and those receiving placebo were clinically significant using a previously defined MCID [24], and the *P*-values were significant. Patients receiving ripretinib had consistently stable self-reported functioning, health status, and QoL. Also, the longitudinal measures suggest these patients were able to maintain QoL, while these same measures declined sharply with placebo after just 1 month.

The importance of evaluating PROs reflective of QoL and function during oncology trials has been increasingly recognized over the past two decades. Whereas TEAEs are determined per investigator assessment, PRO assessments capture the perspectives of patients and can provide insight into the clinical relevance of investigator-determined TEAEs and other AEs that may go undetected during traditional TEAE monitoring. Notably, most of the pivotal trials of imatinib, sunitinib, or regorafenib for the treatment of advanced GIST did not report PROs [35–38]. PRO data are limited for imatinib despite its approval in GIST in 2002, but results derived from phase 3 trials support relatively stable QoL over the course of treatment [39, 40]. In the phase 3 registration trial of regorafenib, PROs were assessed via the EQ-5D 3 Level (EQ-5D-3 L) as an exploratory endpoint and published separately [41]. However, the intent of that analysis was to estimate the health status of patients who were progression-free relative to clinically progressing patients to inform economic models. The study compared baseline and first post-progression scores for both treatment arms combined without providing any insight into changes in EQ-5D-3 L scores with regorafenib relative to placebo or the impact of TEAEs on QoL.

Ripretinib was generally well tolerated in the primary analysis and only 4 patients (4.7%) discontinued study treatment due to a treatment-related TEAE [16]. However, in the INVICTUS study, about 50% of the ripretinib recipients developed some level of alopecia, making it the most common TEAE [16]. Our analyses showed a median time to onset of about 2 months for first detection and worst grade occurrences of alopecia. When stratified by alopecia, patient-reported function, overall health, and overall QoL were generally stable; because patients had already progressed on other therapies and were at risk of fatal progression, perhaps alopecia was not as important to these patients. The impact of hair loss on QoL may depend on many factors, including the proportion of hair lost, the amount of hair the patient had prior to starting treatment, sex, social status, and employment status [42–44]. It is possible that alopecia is not permanent in these patients, but further analysis is necessary.

A limitation of this study was that responses on PRO assessments were not collected from all patients. However, the response rate of approximately 80% in this study is relatively high. Additionally, because these were secondary outcomes, the study arms were not specifically powered for statistical analysis. The number of patients in the placebo group declined quickly due to disease progression, making it difficult to make intergroup comparisons beyond cycle 2. Additional research in real-world patients would be helpful in further establishing the association of alopecia and other symptoms with QoL.

## Conclusion

In conclusion, PRO assessments in the INVICTUS trial demonstrate that patients with advanced GIST on fourth-line or greater therapy maintain QoL and function while receiving ripretinib out to C2D1 compared with patients receiving placebo. Similarly, patients receiving ripretinib were not negatively impacted by alopecia as related to longitudinal QoL and function.

## Supplementary Information

**Additional file** **1:** **Table S1.** EORTC QLQ-C30 change from baseline to C2D1. **Table S2. **EQ-5D-5L scores at baseline and C2D1. **Figure S1.** INVICTUS trial design.

## Data Availability

Qualified scientific and medical researchers can make requests for individual participant data that underlie the results reported in this article, after de-identification, at info@deciphera.com. Proposals for data will be evaluated and approved by Deciphera in its sole discretion. All approved researchers must sign a data access agreement before accessing the data. Data will be available as soon as possible but no later than within 1 year of the acceptance of the article for publication, and for 3 years after article publication. Deciphera will not share data from identified participants or a data dictionary.

## Abbreviations

ANCOVA: analysis of covariance
C: cycle
CI: confidence interval
D: day
ECOG: Eastern Cooperative Oncology Group
EORTC QLQ-C30: European Organisation for Research and Treatment of Cancer Quality of Life Questionnaire
ePRO: electronic PRO
EQ-5D-3L: EuroQoL 5-Dimension 3-Level
EQ-5D-5L: EuroQoL 5-Dimension 5-Level
GIST: gastrointestinal stromal tumor
HR: hazard ratio
MCID: minimal clinically important difference
mRECIST: modified Response Evaluation Criteria In Solid Tumors
NCI-CTCAE: National Cancer Institute Common Terminology Criteria for Adverse Events
OS: overall survival
PD: progressive disease
PDGFRA: platelet-derived growth factor receptor alpha
PFS: progression-free survival
PRO: patient-reported outcome
QoL: quality of life
TEAE: treatment-emergent adverse event
TKI: tyrosine kinase inhibitor
VAS: visual analogue scale
