# Supplementary material for: Patient-reported outcomes in individuals with advanced gastrointestinal stromal tumor treated with ripretinib in the fourth-line setting: analysis from the phase 3 INVICTUS trial
Source: BMC Cancer. 2022 Dec 13;22:1302. doi: 10.1186/s12885-022-10379-9 (PMC9746146; doi:10.1186/s12885-022-10379-9)

**Table S1.** EORTC QLQ-C30 change from baseline to C2D1

|  | **Ripretinib 150 mg QD**  **n = 85** | **Placebo**  **n = 44** |
| --- | --- | --- |
| **Emotional functioning** |  |  |
| Baseline | 70.5 (22.5) | 71.2 (23.51) |
| C2D1 | 76.5 (19.56) | 73.0 (23.94) |
| Change from baseline | 5.1 (16.74) | −2.6 (21.53) |
| **Cognitive functioning** |  |  |
| Baseline | 80.4 (21.78) | 79.8 (21.63) |
| C2D1 | 84.2 (18.85) | 77.3 (26.29) |
| Change from baseline | 2.1 (17.93) | −9.4 (23.16) |
| **Social functioning** |  |  |
| Baseline | 70.5 (28.74) | 67.9 (30.22) |
| C2D1 | 78.9 (23.68) | 72.7 (29.41) |
| Change from baseline | 6.2 (24.27) | −4.7 (29.70) |
| **Fatigue symptoms** |  |  |
| Baseline | 61.0 (27.29) | 60.6 (29.47) |
| C2D1 | 62.3 (24.03) | 54.5 (28.38) |
| Change from baseline | −1.3 (25.49) | −13.2 (25.62) |
| **Nausea/vomiting symptoms** |  |  |
| Baseline | 89.2 (19.20) | 90.5 (18.09) |
| C2D1 | 88.8 (13.54) | 87.4 (25.01) |
| Change from baseline | −2.9 (17.49) | −4.7 (31.46) |
| **Pain symptoms** |  |  |
| Baseline | 65.8 (29.31) | 71.4 (28.82) |
| C2D1 | 70.3 (23.67) | 65.2 (32.64) |
| Change from baseline | 1.0 (27.64) | −10.4 (31.32) |
| **Dyspnea symptoms** |  |  |
| Baseline | 81.1 (21.43) | 78.6 (27.37) |
| C2D1 | 79.3 (23.45) | 82.8 (25.17) |
| Change from baseline | −3.8 (22.37) | −2.1 (25.31) |
| **Insomnia symptoms** |  |  |
| Baseline | 68.5 (32.59) | 72.2 (32.02) |
| C2D1 | 68.8 (28.42) | 63.6 (33.71) |
| Change from baseline | −1.9 (32.54) | −8.3 (32.79) |
| **Appetite loss symptoms** |  |  |
| Baseline | 73.9 (31.34) | 69.8 (37.40) |
| C2D1 | 78.1 (23.80) | 67.7 (35.83) |
| Change from baseline | 1.0 (27.20) | −8.3 (36.91) |
| **Constipation symptoms** |  |  |
| Baseline | 77.5 (27.09) | 80.2 (25.57) |
| C2D1 | 75.1 (29.94) | 74.7 (32.31) |
| Change from baseline | −4.3 (34.01) | −6.3 (26.01) |
| **Diarrhea symptoms** |  |  |
| Baseline | 91.4 (15.66) | 87.3 (22.03) |
| C2D1 | 93.7 (15.17) | 86.9 (29.98) |
| Change from baseline | 1.4 (18.33) | −2.1 (25.31) |
| **Financial difficulty symptoms** |  |  |
| Baseline | 73.0 (31.05) | 69.0 (34.05) |
| C2D1 | 80.6 (25.93) | 70.7 (32.01) |
| Change from baseline | 5.2 (25.15) | −3.1 (19.60) |

All values are mean (SD). C, cycle; D, day; EORTC QLQ-C30, European Organisation for the Research and Treatment of Cancer Quality of Life Questionnaire; QD, once daily.

**Table S2.** EQ-5D-5L scores at baseline and C2D1

|  | **Ripretinib 150 mg QD**  **n = 85** | **Placebo**  **n = 44** |
| --- | --- | --- |
| **Mobility** |  |  |
| **Baseline** |  |  |
| 1 | 41 (48.2) | 25 (56.8) |
| 2 | 18 (21.2) | 8 (18.2) |
| 3 | 12 (14.1) | 6 (13.6) |
| 4 | 2 (2.4) | 2 (4.5) |
| 5 | 1 (1.2) | 1 (2.3) |
| **C2D1** |  |  |
| Patients, n | 79 | 33 |
| 1 | 40 (50.6) | 16 (48.5) |
| 2 | 27 (34.2) | 12 (36.4) |
| 3 | 10 (12.7) | 4 (12.1) |
| 4 | 2 (2.5) | 1 (3.0) |
| **Self-care** |  |  |
| **Baseline** |  |  |
| 1 | 63 (74.1) | 36 (81.8) |
| 2 | 9 (10.6) | 3 (6.8) |
| 3 | 2 (2.4) | 1 (2.3) |
| 4 | 0 | 1 (2.3) |
| 5 | 0 | 1 (2.3) |
| **C2D1** |  |  |
| Patients, n | 79 | 33 |
| 1 | 70 (88.6) | 30 (90.9) |
| 2 | 8 (10.1) | 2 (6.1) |
| 3 | 1 (1.3) | 1 (3.0) |
| **Usual activities** |  |  |
| **Baseline** |  |  |
| 1 | 30 (35.3) | 21 (47.7) |
| 2 | 25 (29.4) | 10 (22.7) |
| 3 | 14 (16.5) | 8 (18.2) |
| 4 | 5 (5.9) | 2 (4.5) |
| 5 | 0 | 1 (2.3) |
| **C2D1** |  |  |
| Patients, n | 79 | 33 |
| 1 | 42 (53.2) | 14 (42.4) |
| 2 | 25 (31.6) | 13 (39.4) |
| 3 | 10 (12.7) | 4 (12.1) |
| 4 | 1 (1.3) | 2 (6.1) |
| 5 | 1 (1.3) | 0 |
| **Pain/discomfort** |  |  |
| **Baseline** |  |  |
| 1 | 18 (21.2) | 10 (22.7) |
| 2 | 36 (42.4) | 18 (40.9) |
| 3 | 14 (16.5) | 9 (20.5) |
| 4 | 6 (7.1) | 4 (9.1) |
| 5 | 0 | 1 (2.3) |
| **C2D1** |  |  |
| Patients, n | 78 | 33 |
| 1 | 18 (23.1) | 9 (27.3) |
| 2 | 36 (46.2) | 11 (33.3) |
| 3 | 20 (25.6) | 9 (27.3) |
| 4 | 3 (3.8) | 4 (12.1) |
| 5 | 1 (1.3) | 0 |
| **Anxiety/depression** |  |  |
| **Baseline** |  |  |
| 1 | 35 (41.2) | 18 (40.9) |
| 2 | 25 (29.4) | 15 (34.1) |
| 3 | 11 (12.9) | 8 (18.2) |
| 4 | 2 (2.4) | 1 (2.3) |
| 5 | 1 (1.2) | 0 |
| **C2D1** |  |  |
| Patients, n | 78 | 33 |
| 1 | 40 (51.3) | 14 (42.4) |
| 2 | 21 (26.9) | 14 (42.4) |
| 3 | 15 (19.2) | 1 (3.0) |
| 4 | 2 (2.6) | 3 (9.1) |
| 5 | 0 | 1 (3.0) |

Data is reported as n (%) unless otherwise indicated.

Each dimension of the EQ-5D-5L has 5 levels: no problems (1), slight problems (2), moderate problems (3), severe problems (4), and extreme problems (5). The digits for the 5 dimensions can be combined into a 5-digit number that describes the patient’s health state.

There were 13 patients with missing information at baseline (11 in the ripretinib arm and 2 in the placebo arm).

C, cycle; D, day; EQ-5D-5L, EuroQoL 5-Dimension 5-Level; QD, once daily.

**Figure S1.** INVICTUS trial design


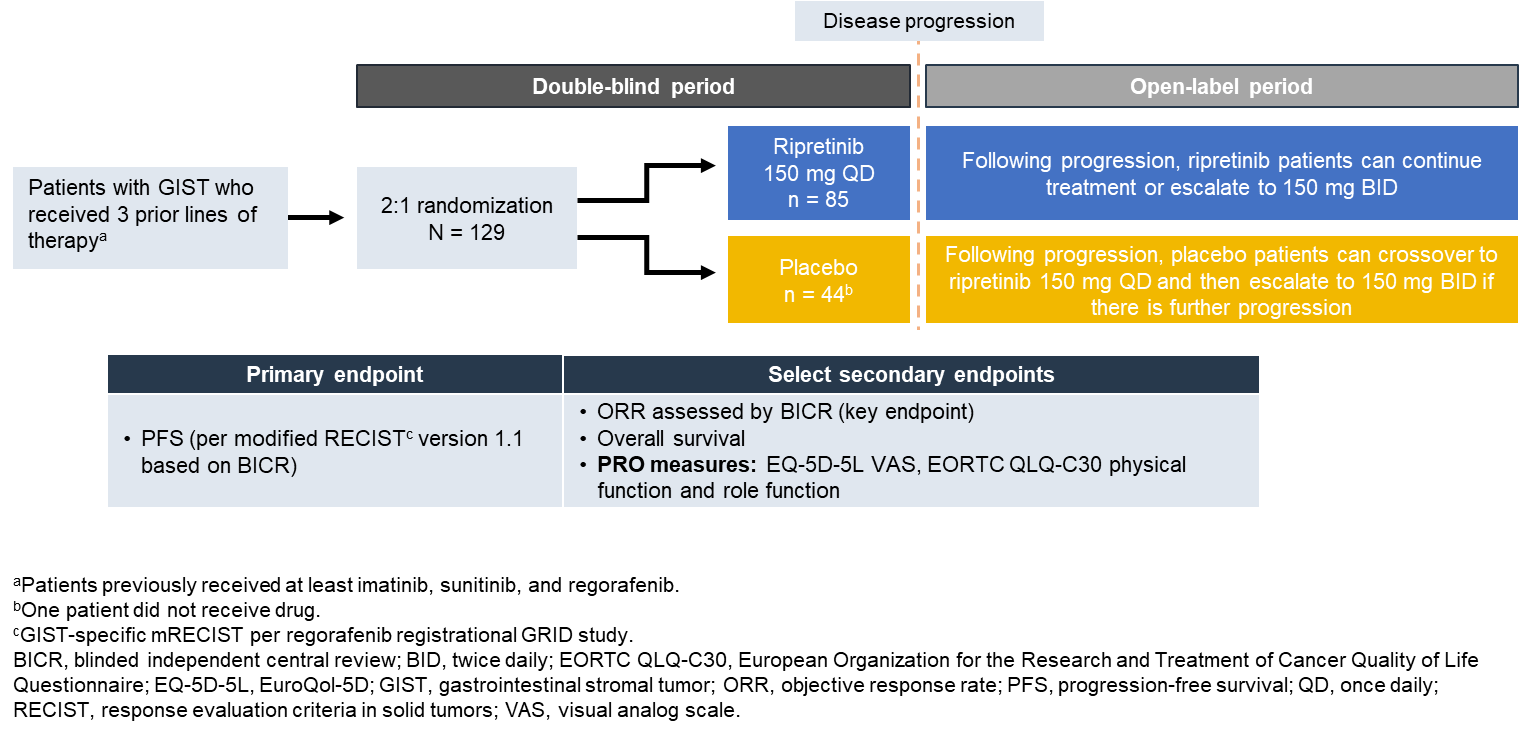

Supplement: Supplementary file 1 — Additional file 1: Table S1. EORTC QLQ-C30 change from baseline to C2D1. Table S2. EQ-5D-5L scores at baseline and C2D1. Figure S1. INVICTUS trial design. [file 12885_2022_10379_MOESM1_ESM.docx]
